# Coronary X-ray angiography segmentation using Artificial Intelligence: a multicentric validation study of a deep learning model

**DOI:** 10.1007/s10554-023-02839-5

**Published:** 2023-04-07

**Authors:** Miguel Nobre Menezes, João Lourenço Silva, Beatriz Silva, Tiago Rodrigues, Cláudio Guerreiro, João Pedro Guedes, Manuel Oliveira Santos, Arlindo L. Oliveira, Fausto J. Pinto

**Affiliations:** 1 Structural and Coronary Heart Disease Unit, Faculdade de Medicina, Cardiovascular Center of the University of Lisbon, Universidade de Lisboa (CCUL@RISE), Av Prof. Egas Moniz, Lisboa, 1649-028 Portugal; 2 Serviço de Cardiologia, Departamento de Coração e Vasos, CHULN Hospital de Santa Maria, Av Prof. Egas Moniz, Lisboa, 1649-028 Portugal; 3 INESC-ID / Instituto Superior Técnico, University of Lisbon, Lisbon, Portugal; 4 Centro Hospitalar de Vila Nova de Gaia, Porto, Portugal; 5 Unidade de Hemodinâmica e Cardiologia de Intervenção, Serviço de Cardiologia, Centro Hospitalar Universitário do Algarve, Hospital de Faro, Faro, Portugal; 6 Unidade de Intervenção Cardiovascular, Serviço de Cardiologia do Centro Hospitalar e Universitário de Coimbra, Praceta Professor Mota Pinto, Coimbra, 3004-561 Portugal; 7 Faculdade de Medicina da Universidade de Coimbra, R. Larga 2, Coimbra, 3000-370 Portugal

**Keywords:** Deep learning, Artificial Intelligence, Machine learning, Coronary angiography, Coronary artery disease, Percutaneous coronary intervention.

## Abstract

**Introduction:**

We previously developed an artificial intelligence (AI) model for automatic coronary angiography (CAG) segmentation, using deep learning. To validate this approach, the model was applied to a new dataset and results are reported.

**Methods:**

Retrospective selection of patients undergoing CAG and percutaneous coronary intervention or invasive physiology assessment over a one month period from four centers. A single frame was selected from images containing a lesion with a 50–99% stenosis (visual estimation). Automatic Quantitative Coronary Analysis (QCA) was performed with a validated software. Images were then segmented by the AI model. Lesion diameters, area overlap [based on true positive (TP) and true negative (TN) pixels] and a global segmentation score (GSS – 0 -100 points) - previously developed and published - were measured.

**Results:**

123 regions of interest from 117 images across 90 patients were included. There were no significant differences between lesion diameter, percentage diameter stenosis and distal border diameter between the original/segmented images. There was a statistically significant albeit minor difference [0,19 mm (0,09–0,28)] regarding proximal border diameter. Overlap accuracy ((TP + TN)/(TP + TN + FP + FN)), sensitivity (TP / (TP + FN)) and Dice Score (2TP / (2TP + FN + FP)) between original/segmented images was 99,9%, 95,1% and 94,8%, respectively. The GSS was 92 (87–96), similar to the previously obtained value in the training dataset.

**Conclusion:**

the AI model was capable of accurate CAG segmentation across multiple performance metrics, when applied to a multicentric validation dataset. This paves the way for future research on its clinical uses.

**Supplementary Information:**

The online version contains supplementary material available at 10.1007/s10554-023-02839-5.

## Introduction

The application of artificial intelligence (AI) to coronary angiography (CAG) has only been ascertained in very few medical/biology publications [1–4]. While the possibilities of such an approach are vast, the first step is arguably to produce accurate segmentation of CAGs, i.e., clearly identifying the coronary tree while excluding other structures.

We have previously published the first results of deep learning models capable of good quality CAG segmentation [5]. In this paper, we aim to validate the results, by applying the model to a new, previously unseen, dataset of coronary angiographies from multiple centers. A well-known validated software was used as reference for segments with non-occlusive lesions, where detailed measurements were undertaken, while also applying the previously described Global Segmentation Score for broad assessment of segmentation quality [5].

## Methods

### Participating centers and equipment

Four centers from across Portugal participated in this study. Images were acquired in Siemens Axiom Artis and Philips Azureon equipment.

### Inclusion criteria

Retrospective selection of consecutive patients who had undergone CAG and percutaneous coronary intervention (PCI) and/or underwent invasive physiology assessment (Fractional Flow Reserve and/or other indexes), within a 1-month period of 2022, regardless of clinical context (i.e. both acute and chronic coronary syndrome). This ensures the model was tested in a real-world context where revascularization was either being considered or performed, thereby excluding a population with normal or near-normal coronary arteries.

### Exclusion criteria

We excluded cases where any of the following applied:

1. Patients with previous cardiac surgery, cardiac devices or other sources of potential artifact.
2. Absence of coronary lesions 50–99% stenosis by visual estimation (i.e. single-vessel ST-elevation myocardial infarction – STEMI - or chronic total occlusions – CTO alone).
3. Poor image quality.
4. Unclear individualization of lesion outline with no overlapping vessels.
5. Unsuccessful automatic measurements with validated software (details below).
6. Unsuccessful software extraction and superimposition of lesion markers on segmented image (details below).

### Image selection

For each selected lesion, a single end-diastolic frame with clear outline definition of the vessel and target lesion was selected. More than one segment per patient and/or image could be used. With an original training dataset of 416 images as previously published [5], we aimed to have a validation dataset of at least 100 images.

### Brief description of previous work and AI model

In our previous work [5] we trained AI models for CAG segmentation using 416 images from patients undergoing physiology or PCI in a single center. The images were manually annotated by a small group (two Cardiology Fellows and an Interventional Cardiologist, who both annotated and supervised the process) and continuously reviewed and corrected, in order to minimize heterogeneity and errors.

We then performed segmentation using an encoder-decoder fully convolutional neural networks based on the U-Net [6], commonly used in medical image segmentation. These are composed of an encoder for extracting image features and a decoder to process those features and produce segmentation masks. To derive the best approach for this task, we conducted a comparative study of encoder and decoder architectures, which resulted in the proposal of the EfficientUNet++, a computationally efficient and high-performing decoder architecture [7], which obtained the best results when combined with an EfficientNet-B5 encoder [8].

To ensure fair evaluation and minimize any bias induced by the input data, each model was tested on data it had not seen during training. The dataset was thus split at the patient level, into 13 subsets of approximately 32 angiograms each. Each subset’s segmentation was performed using a neural network trained exclusively on the remaining data. This enabled the assessment of the segmentation results for the entire cohort, as the usual splitting into a training and testing dataset would have yielded a much smaller group of images for result assessment. The training hyperparameters, namely the number of training epochs and the learning rate decay schedule, were set on the first train-test split, using 1 of the 12 training data subsets for validation. The selected values were then used on every other train-test split, and to train the model on the whole training set of the first split. We also considered cross-validation, but it would be very compute-heavy.

This process resulted in an early AI model, which was then further improved by a second round of manual annotation, where the annotators corrected the resulting imperfections, thereby producing a final training dataset. An “enhanced” model was then trained once again using the same process with the new improved annotated dataset, yielding superior results to the early model, with a final Generalized Dice Score of 93,48/ +/- 2,84%. While we continue to work on improving our model, because the aim of this study is to validate the aforementioned “enhanced” model as previously published [5], no additional training was performed.

### Original images analysis and segmentation

A well-established and validated software (CAAS Workstation 8.5.1) capable of semi-automatic segmentation and Quantitative Coronary Angiography (QCA) was used to generate a reference dataset for comparison. Because it is especially important for a model to correctly segment diseased segments, QCA analysis was performed in selected segments with a stenosis severity of 50–99% by visual estimation. For QCA measurements, calibration was performed either automatically (based on the DICOM information) or by measuring the catheter (5 or 6 Fr), provided it was clearly visible and measurable. The region of interest was then selected and automatic QCA measurements were undertaken.

For each region of interest where successful automatic QCA measurements were undertaken, the lesion diameter, reference diameter, diameter at proximal obstruction border and diameter at distal obstruction border were recorded. The diameter stenosis percentage was calculated as follows: ((reference diameter – lesion diameter) / reference diameter) x 100 [9]. No manual adjustments were accepted, in order to exclude human bias or human-induced imperfection. If the automated outline and measurements were not clearly accurate by visual inspection, the case was excluded (supplementary Fig. 1).

The original images (i.e., without the measurement annotations generated by the CAAS software) were then segmented using our best AI model to date [5], which segments the coronary tree in white and the catheter in red. This process is fully automatic and the only required human input is the image itself. These images were used for testing only, not training.

### Performance assessment

#### Diameters and percentage diameter stenosis

A dedicated python script was written to extract the CAAS markers and superimpose them on the segmentation obtained by the model. The lesion diameter, diameter at proximal obstruction border and diameter at distal obstruction border were then measured using a dedicated python script as well, by verifying the superimposition of the markers with the coronary tree. Because the reference diameter does not exist in the segmented image (which only contains the coronary artery tree and catheter), the CAAS-generated value was used. Percentage stenosis was then calculated using the same equation. Finally, we also compared the measured catheter diameter on the original image versus the segmented image with another adaption of the same script, by measuring the distance between the two parallel lines generated in the original image from the CAAS software. The resulting measurements obtained in the original and the segmented images were then compared.

#### Overlap between original and segmented images

A dedicated python script was also used for assessing the overlap between the original and the segmented images in the region of interest, using the CAAS output as reference. Pixels were then classified as follows:

- True positive (TP): a pixel marked as coronary in both the segmented and original image.
- False positive (FP): a pixel marked as coronary only in the segmented image.
- True negative (TN): a pixel marked as non-coronary in both the segmented and original image.
- False negative (FN): a pixel marked as non-coronary only in the original image.

Using this classification, the following parameters were calculated:

- Accuracy: ([TP + TN]/[TP + TN + FP + FN])
- Sensitivity: TP / (TP + FN).
- Specificity: TN/(TN + FP),
- Positive Predictive Value: TP / (TP + FP).
- Negative predictive value TN/(TN + FN).
- Intersection over Union (IoU): TP / (TP + FN + FP).
- Dice Score: 2TP / (2TP + FN + FP).

#### Global segmentation score

While the above-mentioned criteria offer a detailed account of the model’s accuracy, they do not provide a broad overview of the quality of segmentation as assessed by experts in CAG interpretation (i.e. Cardiologists). As a result, we have previously developed the Global Segmentation Score (GSS), which we have previously applied on the original CAG dataset used to train the AI model (details on its application on supplementary data file) [5]. The GSS was scored by consensus by four Interventional Cardiologists (one from each contributing center).

**Fig. 1:**
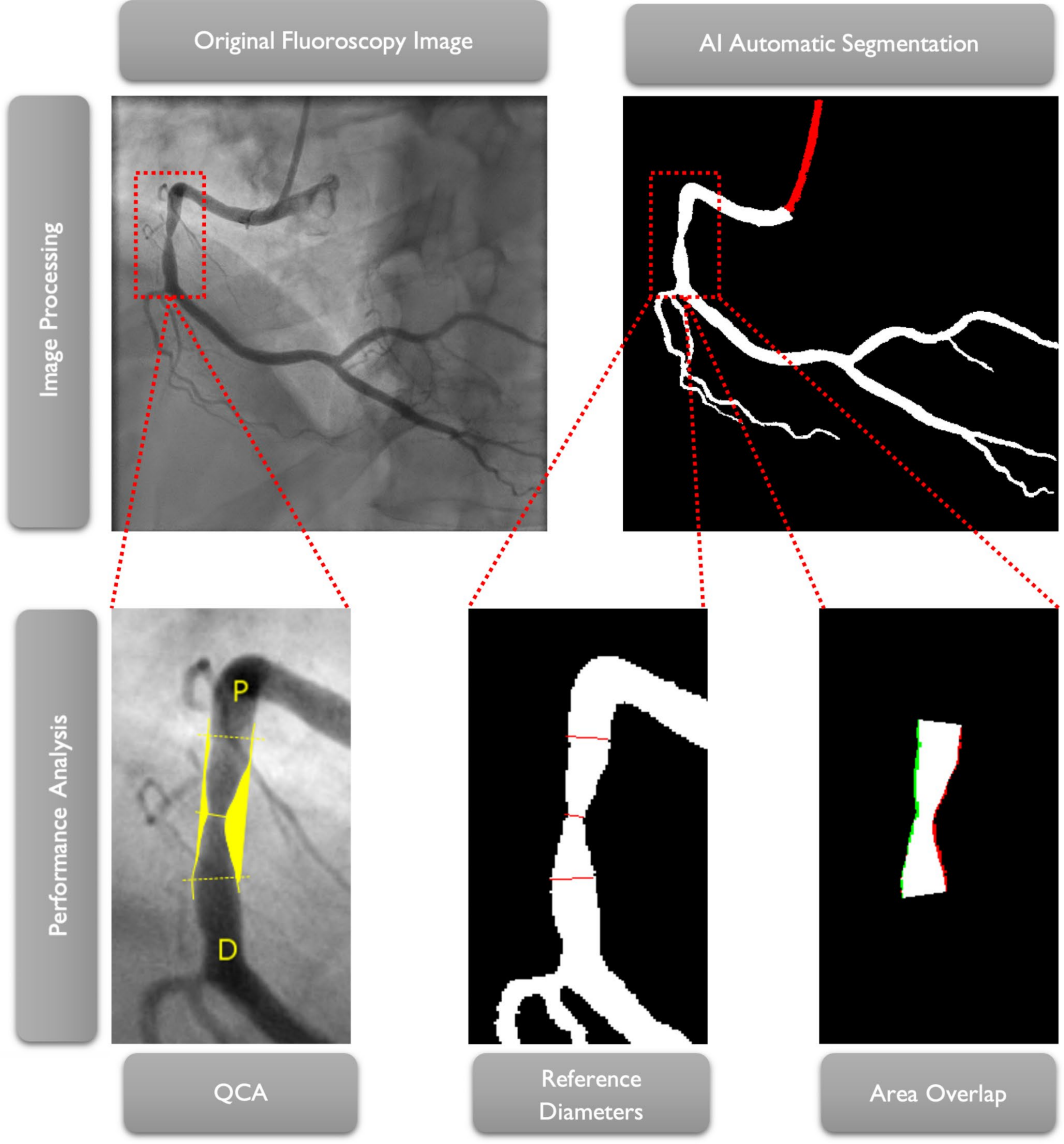
Graphical Abstract: Overview of the segmentation and analysis process. Top left: Baseline CAG of a right coronary artery. Top right: AI automated segmented image. Bottom left: automatic QCA analysis image output in detail. Bottom middle: transposition of the lesion markers on the segmented image in detail. Bottom right: area overlap between the region of interest in the auto-QCA and the segmented image; white pixels are true positives; green pixels are false negatives; red pixels are false positives

Figure 1 summarizes the above-mentioned steps for assessing coronary segmentation.

### Statistical analysis

Descriptive variables are shown in absolute and relative (percentage) numbers. Quantitative variables are shown in average ± standard deviation (if normally distributed) or median (interquartile range) if non-normally distributed. If distribution was normal, we used the paired samples T-test to assess for differences in related samples quantitative variables. If distribution was not normal, we used the Mann-Whitney test (two independent groups) or the Kruskal Wallis test (multiple independent groups) to assess for differences in quantitative variables. A p-value < 0,05 was used for statistical significance. SPSS 27 was used for analysis.

### Ethical issues

This study complies with the Declaration of Helsinki and was approved by the local Ethics’ Institutional Review Board.

## Results

### Baseline characteristics

**Fig. 2:**
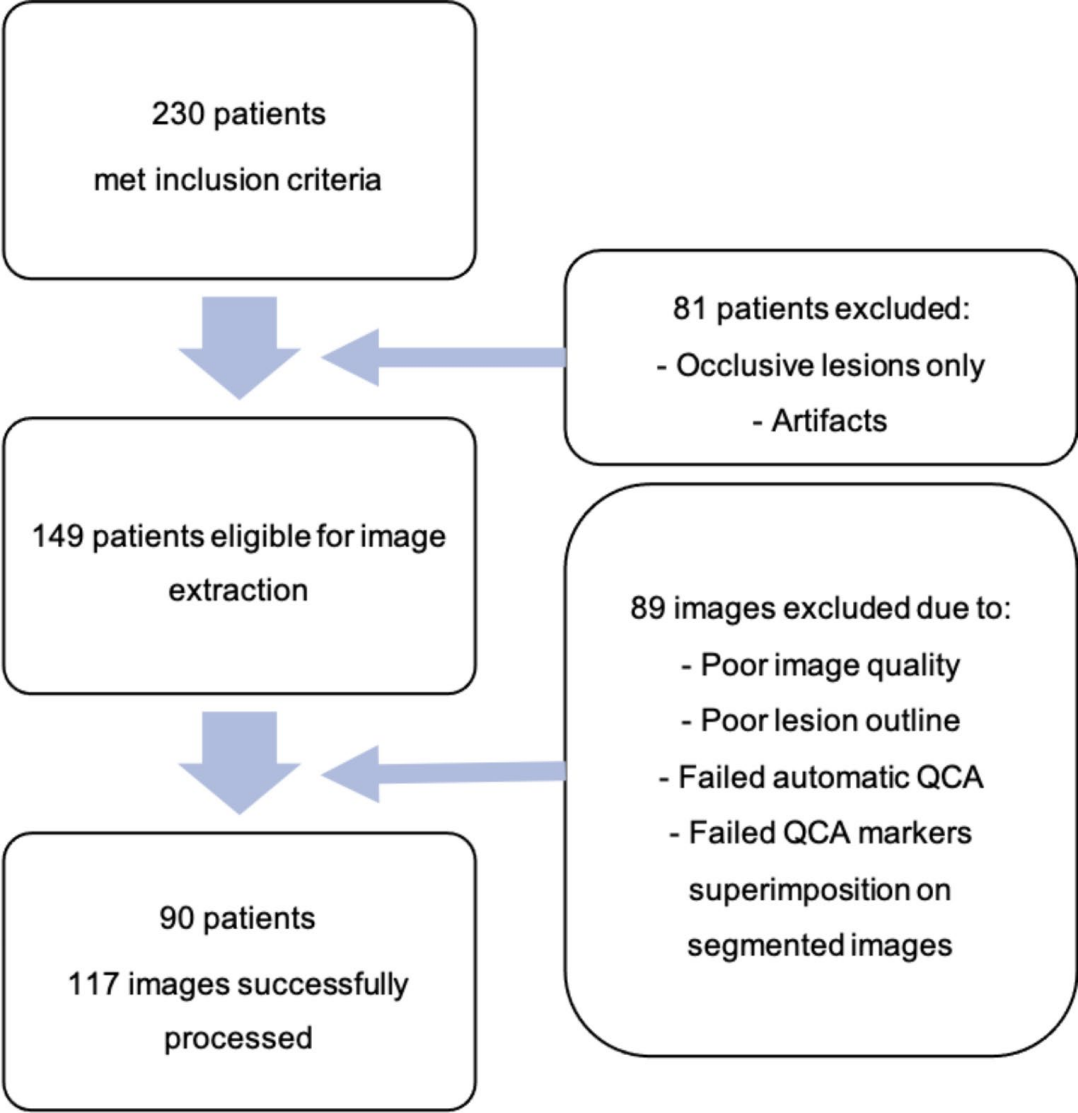
Flowchart of patient and image selection

We included 123 measurements from 117 images, from a total of 90 patients (flowchart in Fig. 2; clinical data on Table 1). The left anterior descending artery (LAD) was the most common target vessel (three measurements were taken on diagonals, two emerging proximally and one emerging in the middle segment of the LAD; all were taken on the proximal segment of the collateral), with measurements taking place more frequently in the middle and proximal segments. As measured by QCA, most lesions had a 50–69% diameter stenosis, with a minority of ≥ 70% lesions (Table 2).

**Table 1:** Clinical characteristics of included patients

Factor	N +/- SD or N(%)
Age	65 +/- 12
Sex (male)	73 (81%)
Hypertension	62 (68,9%)
Diabetes mellitus	28 (31,1%)
Dyslipidemia	55 (61,1%)
Smoker (past or present)	50 (55,6%)
Chronic coronary syndrome	37 (41,1%)
Acute coronary syndrome	53 (58,9%)
Revascularization during/after CAG	76 (84,4%)
Invasive Physiology during procedure	19 (21,1%)

**Table 2:** Distribution of target vessel and lesion severity. LAD: Left Anterior Descending Artery; RCA: Right Coronary Artery; CX: Left Circunflex Artery

Parameter			N (%)
Target Vessel	LAD	Proximal	19
		Middle	23
		Distal	8
		Total	50 (41)
	RCA	Proximal	9
		Middle	24
		Distal	8
		Total	41 (33)
	CX	Proximal	9
		Middle	21
		Distal	2
		Total	32 (26)
Lesion severity		≥ 70%	22 (18)
		50–69%	58 (47)
		< 50%	43 (35)

### Performance

#### Diameters and percentage diameter stenosis

**Fig. 3:**
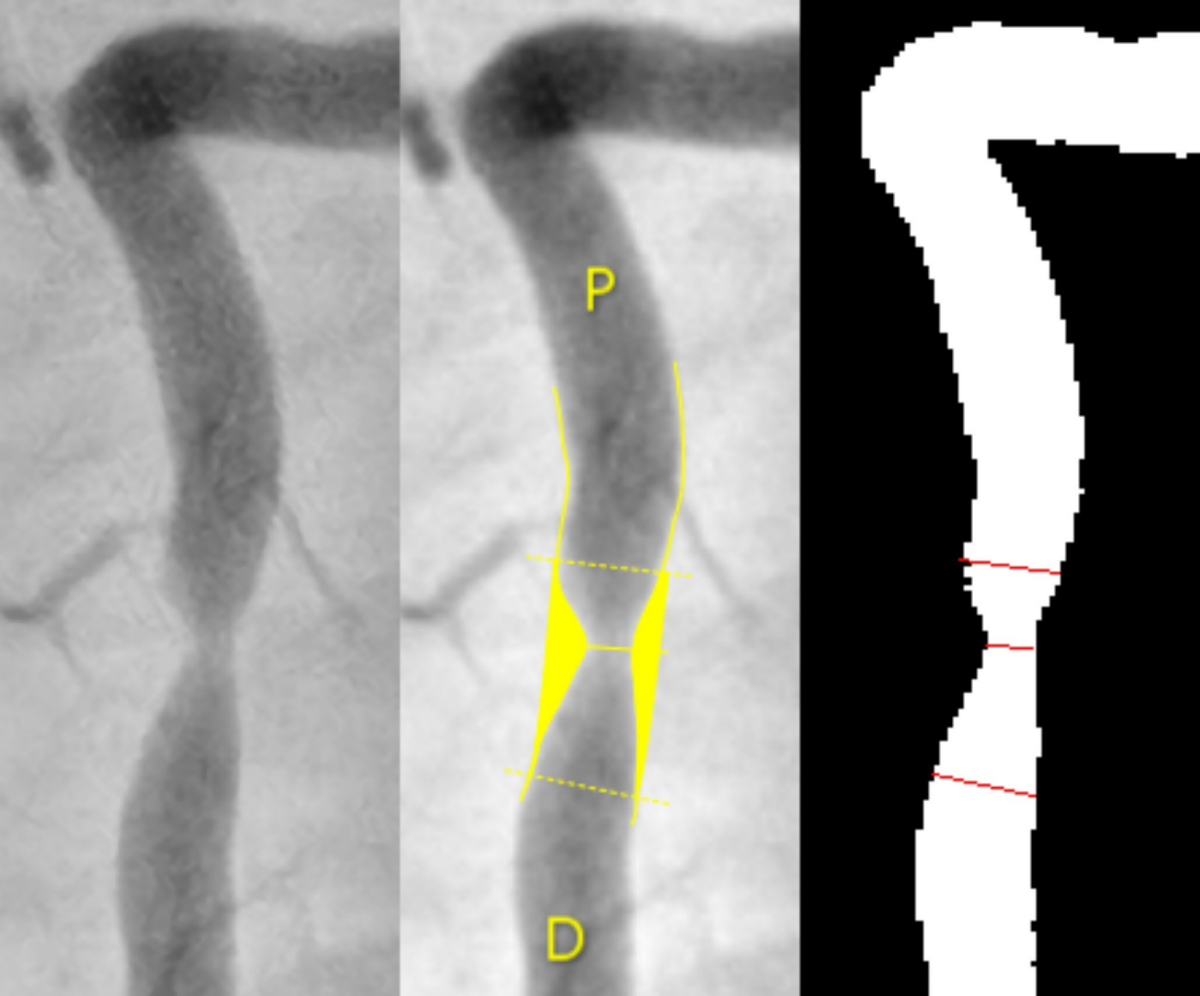
Comparative view of a right coronary artery (56% stenosis by QCA). Left-to-right: original image, auto-QCA, transposition of lines (proximal border diameter, lesion diameter and distal border diameter) to segmented image

Detailed metrics of images (Fig. 3) are depicted in Tables 3 and 4. There were no significant differences for all parameters except for diameter at proximal obstruction border, where the median difference between groups was 0,19 mm. All difference parameters (Table 3) had a non-normal distribution, with the interquartile range demonstrating that there is a clear predominant difference towards the lower-end values, as the 25th quartile is either 0 or very close to 0.

**Table 3:** Detailed measurements between the original and the segmented images. Values shown as mean ± standard deviation. AI – artificial intelligence. *Paired samples T-test;

Parameter	Original Image	AI Generated Segmented Image	P-value*
Diameter Stenosis (%)	56 ± 13	55 ± 13	0,071
Diameter at lesion (mm)	1,06 ± 0,39	1,08 ± 0,37	0,146
Diameter at proximal obstruction border (mm)	2,27 ± 0,54	2,09 ± 0,53	**< 0,01**
Diameter at distal obstruction border (mm)	2,19 ± 0,56	2,15 ± 0,58	0,133

**Table 4:** Median differences between the original and segmented images. Values shown as median (IQ 25th – 75th)

Parameter	Difference
Diameter Stenosis (%)	4,5 (0–7,7)
Diameter at lesion (mm)	0,10 (0–0,17)
Diameter at proximal obstruction border (mm)	0,19 (0,09–0,28)
Diameter at distal obstruction border (mm)	0,10 (0–0,19)

There were no significant differences across stenosis severity (supplementary Table 1) or target vessel (supplementary Table 2). There were also no significant differences considering across centers (supplementary Tables 3 and 4).

**Fig. 4:**
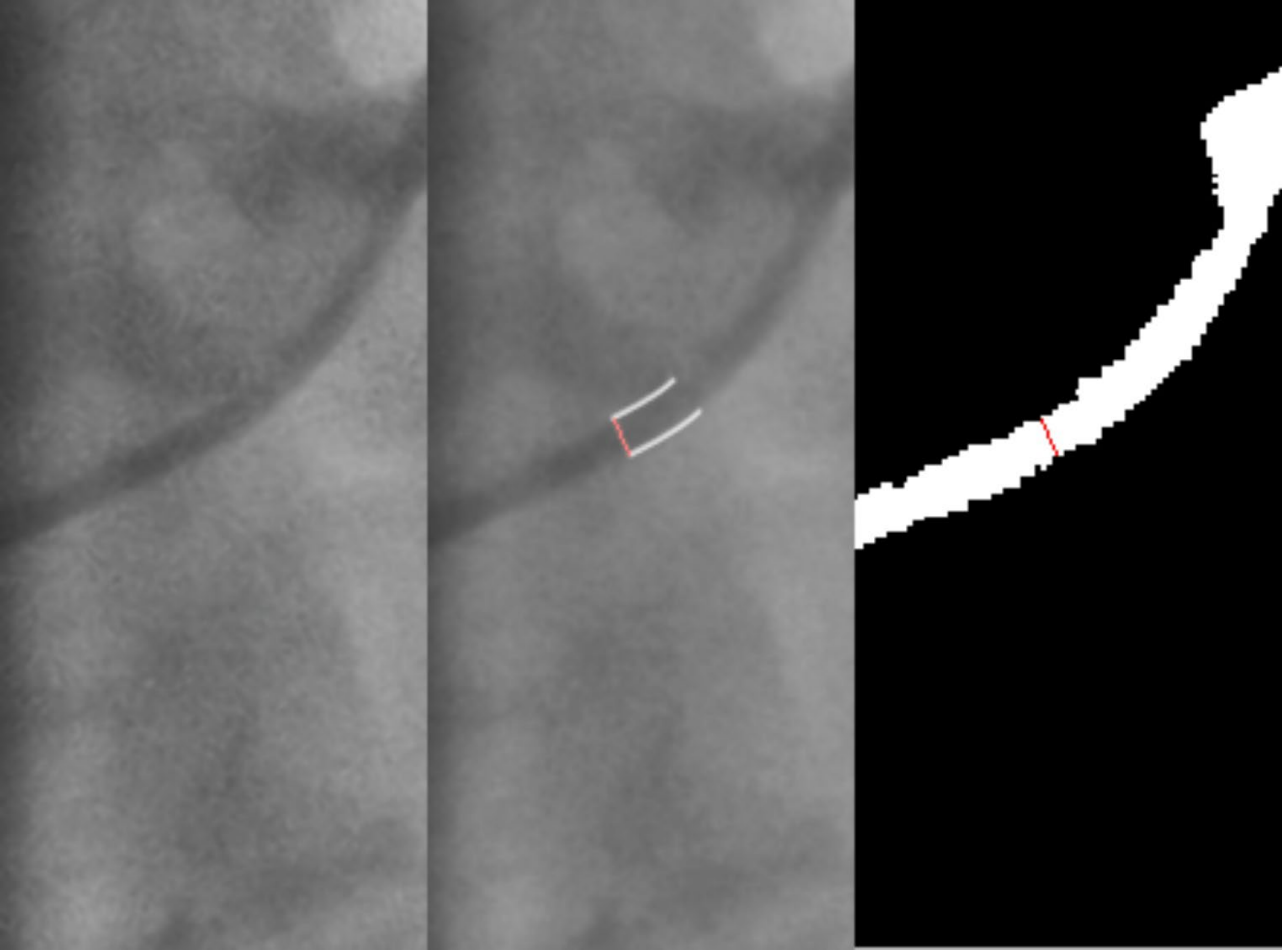
Catheter segmentation assessment. Left-to-right: original image, auto-border detection by reference software, transposition of lines in proximal border to segmented image

With regards to the catheter diameters (Fig. 4), results are shown on supplementary Table 5. A significant number of cases (26/117 − 22%) had to be excluded, either because of collimation (rendering the catheter not visible – 8 cases) or segmentation gaps leading to inaccurate border definition (18 cases). The latter occur because the model focuses especially on segmenting the distal part of the catheter for correctly identifying the transition between catheter and coronary, whereas in the original images calibration occurred predominantly in less distal portions. Because the presence of two groups (5 and 6 Fr) of catheters renders the overall distribution of the sample non-normal, the two groups were analysed separately. There were no significant differences between the original and segmented images. Again, the difference parameter had a non-normal distribution, with the interquartile range demonstrating that there is a clear predominant difference towards the lower-end values.

#### Overlap between original and segmented images

**Fig. 5:**
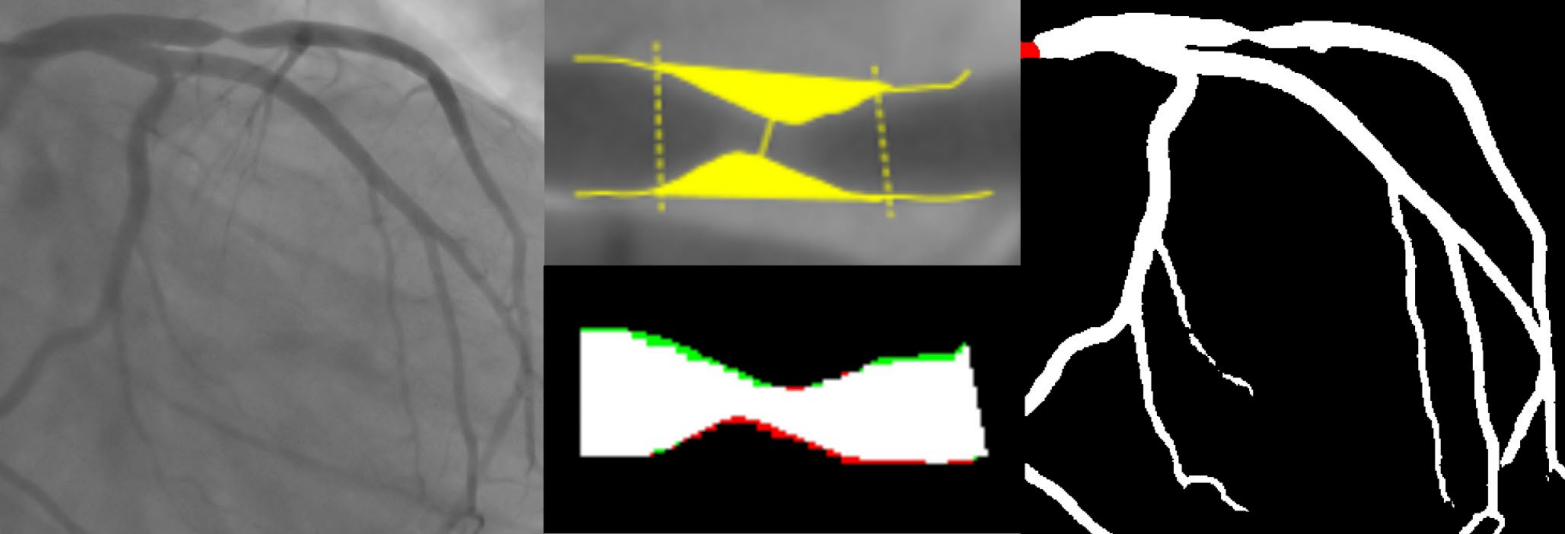
Area overlap in a and left anterior descending 64% stenosis (as measured by QCA).

Results are detailed on Table 5. The model scored ≥ 90% in all metrics (Fig. 5). There were some significant differences between target vessel (supplementary Table 6) and stenosis severity (supplementary Table 7) which, in absolute terms, were between 1 and 3%. There were no differences between centers (supplementary Table 8).

**Table 5:** Overlap metrics. Values shown as median (IQ 25th – 75th)

Accuracy (%)	Sensitivity (%)	Specificity (%)	Positive predictive value (%)	Negative predictive value (%)	Intersection over Union (%)	Dice Score (%)
99,9 (99,9–99,9)	95,1 (92,8–96,4)	99,9 (99,9–99,9)	94,9 (93,1–96,5)	99,9 (99,9–99,9)	90,1 (87,6–91,7)	94,8 (93,4–95,7)

#### Global segmentation score

Results are shown on supplementary Table 9. The model scored well above or close to 90% in most criteria. Catheter gaps were common, usually due to contrast backflow impeding proper visualization of such portions. Catheter artifacts were common and mild gaps in distal parts of small collaterals were quite common as well.

N is lower than overall measurements due to assessment of more than one lesion per image and 8 cases of collimation where the catheter could not be scored, thereby excluding those cases from assessment.

## Discussion

### Main findings

A deep learning AI segmentation model was capable of fully automatic accurate CAG segmentation, as checked by a reference segmentation obtained with validated software and also when assessed by a broad assessment score we previously developed [5].

Diameters at both healthy segments (proximal and distal lesion borders) and diseased segments (diameter at maximum obstruction zone) were similar between the two groups, with statistically significant differences only at the proximal obstruction border. However, in absolute terms, the difference was very small (0,19 mm, a < 10% difference considering the proximal diameter in either group) and we therefore believe it is unlikely to be of clinical significance. The stenosis severity as assessed by percentage stenosis only differed by < 5% in absolute terms, a difference not meaningful either statistically or clinically. The latter is perhaps the single most important finding, as percentage diameter stenosis is the fundamental criteria assessed in clinical practice for proceeding with either revascularization or functional testing, as recommended in current guidelines [10]. Importantly, there were no significant differences in performance regarding target vessel, stenosis severity or centers.

When considering the overlap between the segmented image and the original image, accuracy, specificity and negative predictive value scored close to 100%. This was expected, because most of the image is composed by background rather than artery. As a result, we believe metrics that do not take into account true negatives provide a more faithful indication of the actual model performance. In that regard, sensitivity and positive predictive value still scored quite high, at approximately 95%. The metric that more directly assesses the true overlap between the original and segmented images in the region of interest (correctly identifying all of the vessel while avoiding non-artery pixels) is the intersection over union criteria, which fell just short of 90%. Lastly, the Dice Score puts greater emphasis on the fundamental task of segmentation – correctly identifying the target structure i.e. true positives – in this case, the coronary tree. With an average score of approximately 95%, while also considering all the remaining metrics, we believe our model can be described as accurate. Importantly, the Dice Score in our previous study was 93%, thus very similar to what we now found [5]. There were statistically significant differences in the IoU and Dice Scores between target vessel stenosis severity. Notwithstanding, the absolute differences were very minor (around 1–2%) and therefore of little or no clinical relevance.

With regards to the GSS, our model achieved a high score with a median of 92/100 points, exceedingly similar to what we had previously described in the dataset used to train and develop the model. The model scored very high in almost all tasks, while maintaining minor imperfections with regards to mild gaps in collateral branches, which were very frequent. Catheter segmentation was not as good as coronary segmentation, as usually small catheter artifacts or gaps in the vicinity of the coronary tree origin were common. This was due not only to contrast backflow, but also because of how AI models are trained and function. Indeed, performance is very dependent on class frequency. Because the catheter is a less frequent class (i.e. corresponds to much fewer pixels), the models receive less penalty for errors regarding its segmentation when compared to the coronary tree. This is partly mitigated by the use of an appropriate loss function, but the imbalance nevertheless persists to some extent. Once again, this was very similar to what we saw in the training dataset [5]. With regards to precise catheter measurements, the differences between original and segmented images (for both 5 and 6 Fr catheters) were not statistically significant, suggesting the catheter’s segmentation, from a calliper precision point of view, is accurate. However, due to the above-mentioned limitations and to a small number of images where only a small portion (or none at all) of the catheter was discernible, our sample was somewhat reduced, thereby limiting this assessment.

### Other studies in the field

There are very few studies published in medical/biology journals to date where a comparison with our results can be made. With regards to the GSS in particular, no similar application has ever been undertaken, to our knowledge.

The largest published study [3] included a dataset of 1050 images distributed across all incidences and vessels for performance evaluation. An average 98% accuracy was obtained. While specificity and negative predictive values scored very highly, sensitivity and positive predictive value came closer to 80%. The performance was slightly inferior in more distal vessels. Intersection over union or Dice Score were not reported. Importantly, however, that study’s evaluation used the baseline human annotation as reference, rather than an external validated software, thereby not enabling the identification of bias or imperfections which might have become embedded in their AI model. In our previous study, we demonstrated that even with a small group of annotators and continuous review of the annotations, there is always some degree of imperfection in human annotation [5], hence the relevance of comparing against an automated and validated external software. Additionally, the reported accuracy focuses on the overlap across the entire coronary tree rather than the percentage stenosis of diseased segments. This is advantageous in the sense that a globally accurate performance can be tested. Notwithstanding, we believe testing only for diseased segments actually renders the comparison more demanding. This is because the segmentation of stenotic segments is harder from a technical point of view and also due to the fact that the number of true positive pixels is necessarily smaller in such segments – leading to a lower likelihood of true positives. Whichever interpretation is made, it is clear that an exact comparison with Du et al. [3] is not possible. However, broadly speaking, the accuracy of both models seems quite high and our model seems at least as accurate, if not more.

Su Yang et al. [4] also produced AI models for CAG segmentation. Their validation dataset was somewhat larger (181 images), but their performance seems slightly lower, with all overlap metrics generally scoring just short of 90% and a Dice Score of 89%. Importantly, they also only segmented diseased segments, with a minimum lesion of 30% and used the same reference software as we did. Thus, their results are more directly comparable to ours and our model seems to have superior performance. Two other works [1, 2], from the same baseline dataset, also went on to develop AI-based CAG segmentation, this time with a validation dataset of 550 images. While the model performed well, with an accuracy of 98% and a sensitivity of 87%, they also based their validation dataset on human annotation of the coronary tree without using external software. Thus the above-mentioned considerations for Du et al. [3] also apply.

Recently, Gao et al. [11] published the results of a CAG segmentation model trained on only 130 images. Their methodology, however, is somewhat different, since they combined features from deep learning segmentation models’ features and non-AI image filters to perform pixel-wise classification using gradient-boosting decision trees [12] and deep forests [13]. Their results also show good performance, with a Dice Score of 87,4%, sensitivity of 90,2% and specificity of 99,2%. This highlights that merging deep learning with traditional computer vision methods can yield good results, when working with relatively small datasets. However, no external validation software was used and the whole coronary tree was evaluated. As a result, once more, the previous considerations for Du et al. [3] apply.

Other works in the application of AI to coronary segmentation are primarily technical and featured in engineering publications. A detailed review of these falls outside the scope of this paper and can be consulted in our previous technical publication [7]. However, some considerations regarding these provide further contextualization of our findings.

Xian et al. [14] used a very large dataset of 3200 manually annotated images and experimented with the U-Net architecture as well, with a sensitivity of 90,1%, positive predictive value of 89,8% and Dice Score 90%. However, the annotations were undertaken with a specific software for the purpose of coarsely signaling the vessel route, and focused only on the main vessels. Since we achieved higher performance metrics, it seems a smaller but higher quality dataset, with very precise and cumbersome manual annotations, may be a better approach.

Yang et al [15] have obtained a sensitivity, positive predictive value and Dice Score of 91,3%, 92,5% and 91,9%, respectively, by using popular image classification backbones pre-trained on ImageNet instead of the U-Net’s encoder, while also using a modified generalized dice loss function. Their findings were influential in our training method, as we used a combination of their proposed loss function and the focal loss [16]. Other authors have explored the use of dense connections, improving on the performance of the standard U-Net [17]. This approach is also present in the U-Net ++ [18], which we used in our approach.

In all of the above studies, metrics regarding vessel diameters were not performed. Thus, a direct comparison with this study regarding those is not possible. M’hiri et al. addressed the issue of CAG diameter measurements, when dealing with the issue of diameter variation during the cardiac cycle due to vessel distensibility. They focused mainly in measuring specific segments of the coronary tree, as we did. However, they used a graph-based segmentation method, then tracked the changes across the cardiac cycle using a spatio-temporal segmentation method. They obtained a Dice Score of 98%, with a very small diameter mean error (0,18 mm) [19]. However, they did not focus on diseased regions. While this study is not focused on AI methods, it highlights that other methods may be of use for accurate CAG segmentation, potentially in combination with AI tools [11].

In light of all these studies, the performance of our model seems at least as good, if not better, than previously proposed AI models. We believe this is related to its neural network architecture, which was carefully chosen over a series of experiments [7], taking into consideration the invaluable contributions of previously mentioned studies. In addition to that, we also believe that our manual annotations methodology was essential, as it allowed us to obtain a highly reliable training dataset: a small number of annotators (to reduce heterogeneity) well trained in the interpretation of coronary angiograms; very careful review of annotations with recurrent iterations of quality checks and improvements; and further manual improvement of the already accurate segmentation images produced by an earlier AI model, thus combining the best of AI and human annotations into a final training dataset, as mentioned in the methods section and previous publication [5].

### Limitations

Our study is not without limitations. Despite the multicentric approach, our dataset is relatively small when compared to previously published studies. We also only tested the model performance against validated software in diseased locations, rather than on the whole coronary tree. Therefore, we cannot affirm that the performance would be identical in the remaining areas. However, as previously explained, segmenting zones with lesions is actually more challenging for the model than segmenting broad, mostly healthy segments. In addition to that, we did not find differences regarding target vessel or lesion severity. Plus, considering the results of the GSS, the overall performance regarding CAG segmentation was quite appropriate. Thus, we believe that it is unlikely that performance would be significantly different had we tested for the whole coronary tree. Importantly, if we had chosen to segment whole vessels, it would be very likely that some manual corrections had to be undertaken, which might induce bias or imperfections in the reference images. Hence, the decision to proceed as described was deliberate. The assessment of catheter segmentation was also more limited than that of the coronary tree, as described above.

The exclusion of potential sources of artifacts from devices or previous cardiac surgery means our model is not yet applicable to such patients. Notwithstanding, we didn’t exclude cases with previous implantation of stents, but we did not perform detailed measurements on such segments.

The total number of patients/images who fully met exclusion criteria was somewhat high, thereby limiting the final amount of available images for analysis, which may raise questions as to whether this sample is representative of everyday CAGs and an therefore constitutes an adequate validation dataset. This was the result of somewhat stringent criteria, which we felt were nonetheless necessary due to basic feasibility (such as excluding single-vessel complete occlusion cases where QCA is not applicable, or excluding imaging artifacts for which the models are not yet trained), reduction of bias (such as not allowing for manual QCA correction), or excluding patients with normal/near-normal arteries (where testing would be much less challenging or useful in future clinical application). Notwithstanding, we included patients consecutively rather than selectively and the clinical characteristics of included patients are in agreement with everyday clinical practice. We therefore believe our sample to be reasonably representative of real-world practice. Furthermore, we exceeded the minimum validation target of 100 images, yielding relative rates of training vs. validation cases in agreement with other AI studies [2–4].

The imbalance in sample size limits the comparison between centers.

It has long been established that operators significantly differ in their interpretation of lesion severity and have a tendency to overestimate the importance of a stenosis [20–24], as we also saw in this study. Indeed, while visually all lesions were interpreted as > 50% stenosis, a significant amount of the sample actually had a < 50% stenosis, which further reflects the real-world nature of the dataset.

Lastly, the distance between the 2D centerline and the distance to the closest edge would have also been a good metric for assessing model performance in this setting. We did not perform such testing.

In light of all of the above, concerns may be raised regarding generalization from this dataset. However, we believe that the absence of statistically significant differences across all subgroups at least partially attenuates this concern.

### Future directions

We are currently working in automatic anatomical interpretation, lesion severity based on auto-QCA and integration with physiology. We believe without effective segmentation models, none of these will be possible. Much like for human interpretation of CAG, separating the coronary arteries from everything else in the image is an essential first step. Our ultimate goal is to produce an intelligence augmentation tool that helps physicians perform a more objective and streamlined interpretation of CAG, hopefully contributing for better patient outcomes. As we continuously improve its performance, while also adding new capabilities, clinical application will potentially be possible in the near future, opening a new perspective and potentially more accurate method to assess coronary artery disease.

We are also continuously working to expand and improve the model, as segmentation alone is not a final goal in itself, but rather a fundamental step. We hope to release a public version in the near future, which other researchers may use for whichever application they may deem useful. Importantly, comparing or even merging with future models from other groups may also be very relevant. Since it uses an inherently data-hungry deep learning model, our coronary artery segmentation system would surely benefit from training on a larger volume of data. Manual annotation of coronary angiography images, however, is very cumbersome and time-consuming, and therefore it is difficult to obtain much larger labeled datasets. Hence, significant improvements to the model could probably be achieved, for example, by using self-supervised learning on existing very large volumes of unlabeled data. These possibilities are described in detail in our previous technical publication [7].

### Data Availability

Detailed full-scale study data cannot currently be made publicly available due to limitations imposed by national data protection regulations, as this is a retrospective study and no informed consent was obtainable regarding this particular analysis. Both our research team and others in the national scientific community are working to develop a framework where such would be possible. However, independent replication of our analysis is possible, given that the detailed description of our experimentations and relevant code is publicly available [7].

## Conclusions

Our AI model was capable of accurate CAG segmentation when applied to a multicentric validation dataset, with no differences between target vessels or stenosis severity. This paves the way for future research and implementation for its clinical uses.

## Electronic supplementary material

Below is the link to the electronic supplementary material.

Supplementary Material 1

Supplementary Material 2

Supplementary Fig. 1: Two examples of failed auto-QCA analysis. In the right coronary artery, a subocclusive lesion is visible (upper left image). The software fails to track the lesion accurately (upper right image). In the left anterior descending artery, the software tracks a collateral rather than the main vessel on the left border (original image - bottom left, failed tracking - the bottom right).

## Abbreviations

GSS: Global Segmentation Score
AI: Artificial Intelligence
CAG: Coronary Angiography
STEMI: ST-elevation myocardial infarction
CTO: Chronic Total Occlusion
