# Supplementary material for: Coronary X-ray angiography segmentation using Artificial Intelligence: a multicentric validation study of a deep learning model
Source: Int J Cardiovasc Imaging. 2023 Apr 7;39(7):1385–96. doi: 10.1007/s10554-023-02839-5 (PMC10250252; doi:10.1007/s10554-023-02839-5)
Supplement: Supplementary file 1 — Supplementary Material 1 [file 10554_2023_2839_MOESM1_ESM.docx]

**Global Segmentation Score**

The following 11 criteria are as objectively defined as possible and analysed for each image. Each is independently met or not.

1. **Catheter segmentation**:
   1. **Main segmentation**: The distal part of the catheter (i.e. the closest discernible portion to the coronary artery in the ascending aorta) is correctly segmented and labelled. If minor or non-distal gaps are present, this criterion should be scored as met.
   2. **Gaps** (minor) are absent
   3. **Catheter thickness** is accurate, by visual appreciation
   4. **Location**: if parts of the catheter far from the coronary ostia (ascending and/or descending aorta) are segmented, there are no major gaps or artifacts
2. **Vessel segmentation**:
   1. **Main vessels** are correctly segmented and labelled. For the RCA, this includes the segments from the ostium to the crux. For the LCA, this includes the segments from the left main ostium to the visually discernible distal segments of the left anterior descending or the circumflex (or most important obtuse marginal branch), depending on incidence. Branches are excluded from this criterion. If minor gaps are present, this criterion should be scored as met.
   2. **Branch segmentation**: branches with a luminal diameter of at least approximately 2 mm (using the catheter size as reference) are correctly segmented and labelled. Size is estimated by visual appreciation. If minor gaps are present, this criterion should be scored as met.
   3. **Main vessel gaps** (minor) are absent.
   4. **Branch gaps** (minor) are absent.
   5. Catheter to artery **transition**: correct labelling of the catheter tip vs coronary artery origin.
3. **Artifacts**
   1. **Coronary**: no non-coronary structures are labelled as part of the coronary.
   2. **Catheter**: no non-catheter structures are incorrectly labelled as part of the catheter.

These two artifacts’ criteria are not applicable to the small catheter-artery transition area.

The relevance of each criterion is then weighted as per the following table:

| Criteria | Cathether Vs Coronary  Relative Weight | Individual Criteria  Relative Weight | Points |
| --- | --- | --- | --- |
| Main Vessel Segmentation | 70% | 40% | 28,0 |
| Main Vessel Gaps |  | 10% | 7,0 |
| Catheter to Artery Transition |  | 15% | 10,5 |
| Branch Segmentation |  | 20% | 14,0 |
| BranchGaps |  | 5% | 3,5 |
| Coronary Artifacts |  | 10% | 7,0 |
| Catheter Segmentation | 30% | 40% | 12,0 |
| Catheter Gaps |  | 10% | 3,0 |
| Catheter Artifacts |  | 15% | 4,5 |
| Catheter Location |  | 5% | 1,5 |
| Catheter Thickness |  | 30% | 9,0 |
| Total |  |  | 100 |
