# Supplementary material for: Coronary X-ray angiography segmentation using Artificial Intelligence: a multicentric validation study of a deep learning model
Source: Int J Cardiovasc Imaging. 2023 Apr 7;39(7):1385–96. doi: 10.1007/s10554-023-02839-5 (PMC10250252; doi:10.1007/s10554-023-02839-5)
Supplement: Supplementary file 2 — Supplementary Material 2 [file 10554_2023_2839_MOESM2_ESM.docx]

Supplementary table 1: differences in diameter stenosis (%), lesion diameter, proximal obstruction border diameter and distal obstruction border diameter per stenosis severity. Values shown as median (IQ 25^th^ – 75th). *Kruskal-Wallis test

| Stenosis Severity - original QCA image as reference (N, %) | Diameter stenosis (%) difference | Diameter at lesion (mm) difference | Diameter at proximal obstruction border (mm) difference | Diameter at distal obstruction border (mm) difference |
| --- | --- | --- | --- | --- |
| ≥ 70% (22, 18%)) | 4,9 (2,2 – 8,3) | 0,12 (0,06 – 0,18) | 0,13 (0,08 – 0,27) | 0,15 (0,06 – 0,27) |
| 50-69%(58, 47%) | 5,2 (0 – 7,7) | 0,12 (0 - 0,18) | 0,18 (0,10 – 0,31) | 0,10 (0 – 0,20) |
| < 50% (43, 35%) | 4,7 (0 – 6,2) | 0,08 (0 – 0,15) | 0,16 (0,10 – 0,27) | 0,08 (0 – 0,15) |
| P-value* | 0,224 | 0,274 | 0,651 | 0,094 |

Supplementary table 2: differences in diameter stenosis (%), lesion diameter, proximal obstruction border diameter and distal obstruction border diameter per target vessel. Values shown as median (IQ 25^th^ – 75th). *Kruskal-Wallis test; LAD – Left Anterior Descending; RCA; Right Coronary Artery; CX: Circumflex Artery

| Target Vessel | Diameter stenosis (%) difference | Diameter at lesion (mm) difference | Diameter at proximal obstruction border (mm) difference | Diameter at distal obstruction border (mm) difference |
| --- | --- | --- | --- | --- |
| LAD | 4,7 (0 – 7,8) | 0,09 (0 – 0,17) | 0,17 (0,09 – 0,32) | 0,11 (0 – 0,19) |
| RCA | 4,1 (0 – 7,7) | 0,11 (0 - 0,20) | 0,17 (0,08 – 0,28) | 0,11 (0 – 0,20) |
| CX | 4,2 (0 – 6,8) | 0,09 (0 – 0,15) | 0,15 (0,10 – 0,29) | 0,08 (0 – 0,18) |
| P-value* | 0,801 | 0,817 | 0,569 | 0,679 |

Supplementary table 3: detailed measurements between the original and the segmented images per center. Values shown as mean ± standard deviation. AI – artificial intelligence. *Paired samples T-test;

| Center (N/%) | Parameter | Original Image | AI Generated Segmented Image | P-value* |
| --- | --- | --- | --- | --- |
| A (39 / 32%) | Diameter Stenosis (%) | 60 ± 11 | 59 ± 11 | 0,203 |
|  | Diameter at lesion (mm) | 0,88 ± 0,33 | 0,91 ± 0,33 | 0,261 |
|  | Diameter at proximal obstruction border (mm) | 2,14 ± 0,49 | 1,99 ± 0,45 | **< 0,01** |
|  | Diameter at distal obstruction border (mm) | 2,04 ± 0,46 | 2,04 ± 0,53 | 0,930 |
| B (36 / 29%) | Diameter Stenosis (%) | 54 ± 14 | 53 ± 12 | 0,223 |
|  | Diameter at lesion (mm) | 1,13 ± 0,39 | 1,16 ± 0,34 | 0,277 |
|  | Diameter at proximal obstruction border (mm) | 2,38 ± 0,58 | 2,17 ± 0,61 | **< 0,01** |
|  | Diameter at distal obstruction border (mm) | 2,27 ± 0,61 | 2,22 ± 0,61 | 0,067 |
| C (26 / 21%) | Diameter Stenosis (%) | 54 ± 14 | 54 ± 15 | 0,818 |
|  | Diameter at lesion (mm) | 1,22 ± 0,40 | 1,24 ± 0,42 | 0,778 |
|  | Diameter at proximal obstruction border (mm) | 2,49 ± 0,49 | 2,28 ± 0,48 | **< 0,01** |
|  | Diameter at distal obstruction border (mm) | 2,47 ± 0,52 | 2,39 ± 0,60 | 0,170 |
| D (22 / 18%) | Diameter Stenosis (%) | 53 ± 12 | 52 ± 13 | 0,504 |
|  | Diameter at lesion (mm) | 1,06 ± 0,33 | 1,07 ± 0,31 | 0,842 |
|  | Diameter at proximal obstruction border (mm) | 2,07 ± 0,52 | 1,88 ± 0,53 | **< 0,01** |
|  | Diameter at distal obstruction border (mm) | 1,99 ± 0,53 | 1,97 ± 0,55 | 0,648 |

Supplementary table 4: differences in diameter stenosis (%), lesion diameter, proximal obstruction border diameter and distal obstruction border diameter per center. Values shown as median (IQ 25^th^ – 75th). *Kruskal-Wallis test

| Center (N / %) | Diameter stenosis (%) difference | Diameter at lesion (mm) difference | Diameter at proximal obstruction border (mm) difference | Diameter at distal obstruction border (mm) difference |
| --- | --- | --- | --- | --- |
| A (39 / 32%) | 5,0 (0 – 6,9) | 0,11 (0 - 0,17) | 0,11 (0,08 – 0,27) | 0,09 (0 – 0,17) |
| B (36 / 29%) | 3,4 (0 – 7,8) | 0,09 (0 – 0,20) | 0,19 (0,09 – 0,32) | 0,09 (0 – 0,17) |
| C (26 / 21%) | 4,0 (0 – 7,7) | 0,09 (0 – 0,19) | 0,24 (0,10 – 0,28) | 0,10 (0 – 0,21) |
| D (22 / 18%) | 4,7 (0 – 7,7) | 0,11 (0 – 0,16) | 0,16 (0,10 – 0,28) | 0,13 (0 – 0,23) |
| P-value* | 0,635 | 0,952 | 0,354 | 0,527 |

Supplementary table 5: differences in catheter dimensions. *Mean ± Standard Deviation ** median (IQ 25^th^ – 75th) *** Paired samples T-test AI – artificial intelligence.

| Size | Parameter | Original Image | AI Generated Segmented Image | P-value*** |
| --- | --- | --- | --- | --- |
| 5 Fr (78 cases) | Diameter* (mm) | 1,79 ± 0,08 | 1,77 ± 0,14 | 0,091 |
|  | Difference** (mm) | 0,04 (0 – 0,13) |  |  |
| 6 Fr (13 cases) | Diameter* | 2,05 ± 0,08 | 2,1 ± 0,3 | 0,270 |
|  | Difference** | 0,16 (0 – 0,76) |  |  |

Supplementary table 6: differences in overlap metrics per target vessel. Values shown as median (IQ 25^th^ – 75th). *Kruskal-Wallis test ; LAD – Left Anterior Descending; RCA; Right Coronary Artery; CX: Circumflex Artery

| Target Vessel | Accuracy (%) | Sensitivity (%) | Specificity (%) | Positive predictive value (%) | Negative predictive value (%) | Intersection over Union (%) | Dice Score (%) |
| --- | --- | --- | --- | --- | --- | --- | --- |
| LAD | 99,9 (99,9 – 99,9) | 94,2 (91,8 – 96,2) | 99,9 (99,9 – 99,9) | 94,6 (91,9 – 95,9( | 99,9 (99,9 – 99,9) | 89,1 (85,5 – 90,1) | 94,2 (92,2 – 95,0) |
| RCA | 99,9 (99,9 – 99,9) | 95,1 (92,9 – 96,6) | 99,9 (99,9 – 99,9) | 95,1 (93,6 – 96,6) | 99,9 (99,9 – 99,9) | 90,8 (88,1 – 92,0) | 95,2 (93,7 – 95,9) |
| CX | 99,9 (99,9 – 99,9) | 95,5 (94,3 – 96,4) | 99,9 (99,9 – 99,9) | 95,2 (93,4 – 96,6) | 99,9 (99,9 – 99,9) | 91,9 (90,1 – 93,5) | 96,8 (96,3 – 96,9) |
| P-value* | 0,176 | 0,386 | 0,140 | 0,119 | 0,412 | **0,01** | **0,01** |

Supplementary table 7: differences in overlap metrics per stenosis severity. Values shown as median (IQ 25^th^ – 75th). *Kruskal-Wallis test

| Stenosis Severity (original image as reference) | Accuracy (%) | Sensitivity (%) | Specificity (%) | Positive predictive value (%) | Negative predictive value (%) | Intersection over Union (%) | Dice Score (%) |
| --- | --- | --- | --- | --- | --- | --- | --- |
| ≥ 70% | 99,9 (99,9 – 99,9) | 95,2 (91,8 – 96,6) | 99,9 (99,9 – 99,9) | 95,2 (91,8 – 97,5) | 99,9 (99,9 – 99,9) | 89,3 (84,4 – 91,6) | 94,3 (91,6 – 95,6) |
| 50-69% | 99,9 (99,9 – 99,9) | 95,3 (92,3 – 96,5) | 99,9 (99,9 – 99,9) | 94,6 (91,8 – 96,0) | 99,9 (99,9 – 99,9) | 89,3 (85,5 – 91,4) | 94,3 (92,2 – 95,5) |
| < 50% | 99,9 (99,9 – 99,9) | 95,0 (94,2 – 96,2) | 99,9 (99,9 – 99,9) | 95,6 (94,6 – 96,6) | 99,9 (99,9 – 99,9) | 90,1 (89,2 – 91,9) | 95,0 (94,3 – 95,8) |
| P-value* | 0,482 | 0,969 | 0,376 | **0,024** | 0,581 | **0,029** | **0,029** |

Supplementary table 8: overlap metrics per center. Values shown as median (IQ 25^th^ – 75th).

| Center (N / %) | Accuracy (%) | Sensitivity (%) | Specificity (%) | Positive predictive value (%) | Negative predictive value (%) | Intersection over Union (%) | Dice Score (%) |
| --- | --- | --- | --- | --- | --- | --- | --- |
| A (39 / 32%) | 99,9 (99,9 – 99,9) | 95,4 (93,3 – 96,6) | 99,9 (99,9 – 99,9) | 94,7 (92,3 – 96,8) | 99,9 (99,9 – 99,9) | 89,3 (87,8 – 92,1) | 94,4 (93,5 – 95,9) |
| B (36 / 29%) | 99,9 (99,9 – 99,9) | 94,9 (93,2 – 96,4) | 99,9 (99,9 – 99,9) | 94,6 (92,9 – 96,1) | 99,9 (99,9 – 99,9) | 90,4 (86,7 – 91,9) | 94,9 (92,9 – 95,8) |
| C (26 / 21%) | 99,9 (99,9 – 99,9) | 95,0 (92,2 – 96,1) | 99,9 (99,9 – 99,9) | 95,6 (94,9 – 96,8) | 99,9 (99,9 – 99,9) | 90,6 (89,4 – 92,1) | 95,0 (94,4 – 95,9) |
| D (22 / 18%) | 99,9 (99,9 – 99,9) | 94,8 (92,6 – 96,2) | 99,9 (99,9 – 99,9) | 94,6 (91,5 – 95,6) | 99,9 (99,9 – 99,9) | 89,3 (84,7 – 90,7) | 94,4 (91,7 – 95,1) |
| P-value* | 0,756 | 0,725 | 0,516 | 0,059 | 0,816 | 0,168 | 0,168 |

Supplementary table 9: GSS and individual parameter scores. Median (IQR) in bold due to non-normal distribution.

| Parameter | Criteria |
| --- | --- |
| GSS (Median (IQ 25^th^ – 75^th^)) | **92 (87 – 96)** |
| GSS (Mean ± Standard Deviation) | 90 ± 8 |
| Main Vessel Segmentation (N / %) | 108 (99,1) |
| Main Vessel Gaps (N / %) | 104 (95,4) |
| Catheter to Vessel Transition (N / %) | 103 (94,5) |
| Branch Segmentation (N / %) | 101 (92,7) |
| Branch Gaps (N / %) | 28 (25,7) |
| Coronary Artifacts (N / %) | 95 (87,2) |
| Catheter Segmentation (N / %) | 106 (97,2) |
| Catheter Gaps (N / %) | 78 (71,6) |
| Catheter Artifacts (N / %) | 52 (47,7) |
| Catheter Location (N / %) | 105 (96,3) |
| Catheter Thickness (N / %) | 104 (95,4) |
