# Supplementary figures and images for: Coronary X-ray angiography segmentation using Artificial Intelligence: a multicentric validation study of a deep learning model
Source: Int J Cardiovasc Imaging. 2023 Apr 7;39(7):1385–96. doi: 10.1007/s10554-023-02839-5 (PMC10250252; doi:10.1007/s10554-023-02839-5)

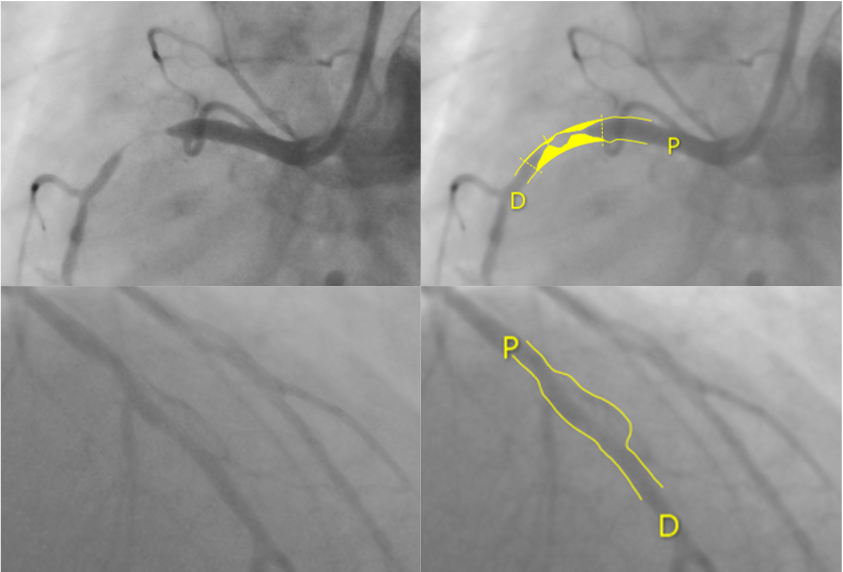

Supplement: Supplementary file 3 — Supplementary Fig. 1: Two examples of failed auto-QCA analysis. In the right coronary artery, a subocclusive lesion is visible (upper left image). The software fails to track the lesion accurately (upper right image). In the left anterior descending artery, the software tracks a collateral rather than the main vessel on the left border (original image - bottom left, failed tracking - the bottom right). [file 10554_2023_2839_MOESM3_ESM.png]
